# LC3B Binds to the Autophagy Protease ATG4b with High Affinity Using a Bipartite Interface

**DOI:** 10.1021/acs.biochem.2c00482

**Published:** 2022-10-20

**Authors:** Yinyan Tang, Amber Kay, Ziwen Jiang, Michelle R. Arkin

**Affiliations:** ^†^Department of Pharmaceutical Chemistry and ^‡^Small Molecule Discovery Center, University of California, San Francisco, California 94158, United States

## Abstract

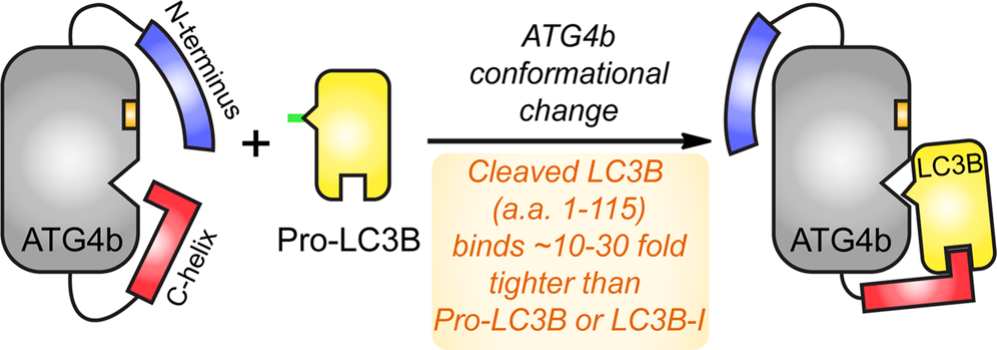

Autophagy is a catabolic cellular process in which unwanted proteins and organelles are degraded by lysosomes. It is characterized by the formation of the double-membrane autophagosome decorated with LC3B, a protein that mediates autophagosomal fusion with lysosomes. The cysteine protease ATG4b acts at two stages in the life cycle of LC3B. We set out to characterize the protein–protein interaction between LC3B and ATG4b. Through biochemical and biophysical studies, we show that the ubiquitin-like core of LC3B (residues 1–115; “LC3B-115”), which lacks the C-terminal cleavage site (between residue 120 and 121), binds to full-length ATG4b with a surprisingly tight dissociation constant (*K*_D_) in the low nanomolar range; 10–30-fold tighter than that of the substrate pro-LC3B (residues 1–125) or the product LC3B-I (residues 1–120). Consequently, LC3B-115 is a potent inhibitor of the ATG4b-mediated cleavage of pro-LC3B (IC_50_ = 15 nM). Binding of the LC3B-115 has no effect on the conformation of the active site of ATG4b, as judged by the turnover of a peptide substrate (“substrate-33”), derived from LC3B-I residues 116–120. Conversely, truncations of ATG4b show that binding and proteolysis of LC3B critically depend on the C-terminal tail of ATG4b, whereas proteolysis of the peptide substrate-33 does not require the C-terminal tail of ATG4b. These results support a bipartite model for LC3B-ATG4b binding in which the core of LC3B binds to ATG4b and the C-terminal tail of pro-LC3B organizes the ATG4b active site; additionally, the C-terminal tail of ATG4b contributes at least 1000-fold higher binding affinity to the LC3B-ATG4b interaction and likely wraps around the LC3B-ubiquitin core. PPIs are often described as containing an energetic “hot spot” for binding; in the case of LC3B-ATG4b, however, the substrate–enzyme complex contains multiple, energetically relevant domains that differentially affect binding affinity and catalytic efficiency.

## Introduction

The activities of proteases are highly regulated in cells, and misregulation of the activity of proteases is associated with diseases.^1^ Many proteases require exosites—non-active site interaction surfaces—to achieve selectivity and catalytic efficiency toward their physiological substrates.^2^ For example, the heparin-binding exosites of thrombin have been extensively studied, leading to the development of heparin-based drugs for blood clotting.^3,4^ Therefore, characterization of exosite interactions can provide valuable insights to better understand the physiological roles of the proteases in biological pathways and to design and develop protease inhibitors or activators as therapeutic agents.

The cysteine protease ATG4b and its substrate LC3B play a central role in the biogenesis of the autophagosome,^5,6^ which is essential in an evolutionarily conserved catabolic process called autophagy.^7−10^ LC3B is synthesized as a cytoplasmic precursor (Pro-LC3B, residues 1–125) containing a ubiquitin-like core (residues 1–115) and a 10-residue C-terminal tail (residues 116–125) that is cleaved near its C-terminus by ATG4 to generate LC3B-I (residues 1–120).^11^ The C-terminal glycine residue of LC3B-I is then conjugated to phosphatidyl ethanolamine, generating membrane-bound LC3B-II on the autophagosome.^12,13^ LC3B-II is delipidated by ATG4 to recycle LC3B-I and enable further autophagosome biogenesis.^14,15^ Among the four human ATG4 homologs, ATG4b has the highest catalytic efficiency for cleaving the C-terminus of LC3B in biochemical studies.^16−18^ Moreover, knockdown of ATG4b shows a clear reduction in basal- and starvation-induced autophagy in mice.^19^ Thus, ATG4b plays dual roles in the processing of LC3B during autophagy. Due to the role of autophagy in cancer-cell survival, ATG4b is being pursued as a drug target for cancer therapy.^20,21^

Several synthetic peptide substrates have been developed for ATG4b. One such peptide substrate called “substrate-33” is derived from LC3B 116–120.^22^ These peptide tools are well suited for studying substrate recognition in the active site and for screening small-molecule modulators. However, the catalytic efficiency of ATG4b for those peptide substrates is much lower than that of natural protein substrates such as LC3B,^23^ indicating that ATG4b might have exosites that have not yet been characterized. Indeed, the X-ray crystal structures of apo-ATG4b and its complex with LC3B reveal that ATG4b changes conformation in two distinct domains upon LC3B binding.^14^ Near the active site, the N-terminal tail of ATG4b moves to unmask the substrate-binding S’ end of the active site,^24^ and a regulatory loop that blocks the entrance of the active site of ATG4b is displaced by LC3B residues 116–125.^25^ Less is known about the conformation of the C-terminal end of ATG4b because the last 39 residues (355–393) of ATG4b are truncated in the ATG4b/LC3B co-structure; however, the C-terminal tail of ATG4b in the apo structure overlaps with the binding site of LC3B, suggesting conformational changes in the ATG4b C-terminus upon binding. Indeed, the deletion of the C-terminal 39 residues of *Xenopus laevis* ATG4b diminishes its binding to the substrate,^26^ and the last several residues (the LC3B interaction region, or LIR) contribute significantly to substrate binding.^27^ Phosphorylation of Ser-383 and Ser-392 in the C-terminal tail of human ATG4b has been shown to modulate autophagy by increasing the delipidation activity of ATG4b on LC3B-II,^28^ which further underscores the importance of the C-terminal tail of ATG4b. However, to our knowledge, no quantitative analysis of the protein–protein interaction (PPI) between ATG4b and LC3B has been reported.

**Scheme 1:**
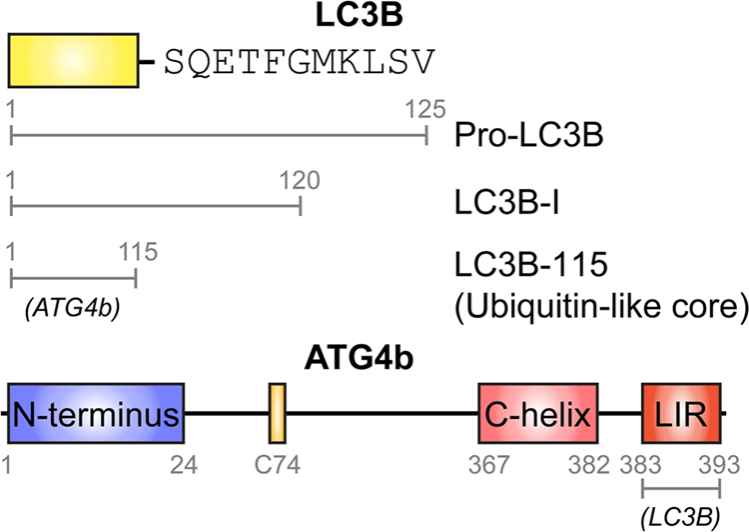
Domains and Interaction Sites of LC3B and ATG4b The interacting partner is denoted in italics.

To develop a model of how ATG4b binds its substrate LC3B, we prepared several truncations of both LC3B and ATG4b and measured the effect of truncations on binding affinity and catalytic efficiency (Scheme 1). Interestingly, the shortest truncation of LC3B (LC3B-115), which only contained the LC3B ubiquitin-like core, had a surprisingly tight-binding affinity for ATG4b [*K*_D_ ∼9 nM by surface plasmon resonance (SPR)]. In support of this novel finding, LC3B-115 was also a 15 nM inhibitor of ATG4b-mediated hydrolysis of pro-LC3B. Binding of LC3B-115 had no effect on the catalytic efficiency of ATG4b’s cleavage of peptide substrate-33, suggesting that the LC3B core domain did not impact the conformation of the ATG4b active site. We also prepared C-terminal truncations of ATG4b that ended at residues 354 (the construct used in the ATG4b-LC3B co-structure), 366 and 382, respectively. In enzymatic assays, ATG4b-354 and ATG4b-366 retained full catalytic activity toward the peptide substrate but lost essentially all activity toward the full-length pro-LC3B substrate. These data are consistent with the loss of binding to LC3B but the maintenance of the active site itself, adding quantitative insights into the impact of the C-terminal LC3-interacting region (LIR) on binding and enzymology. Taken together, these studies support a model in which LC3B binds to ATG4b using both the ubiquitin-like core and the C-terminal tail of LC3B in a bipartite interaction. The C-terminal tail of ATG4b is responsible for high-affinity binding of the core LC3B, and this interface represents the exosite of ATG4b. The ubiquitin-like core of LC3B could serve as an inhibitor itself or could inspire the design of peptidomimetic inhibitors for autophagy.

## Results

### Design of LC3B Truncation Constructs

**Figure 1:**
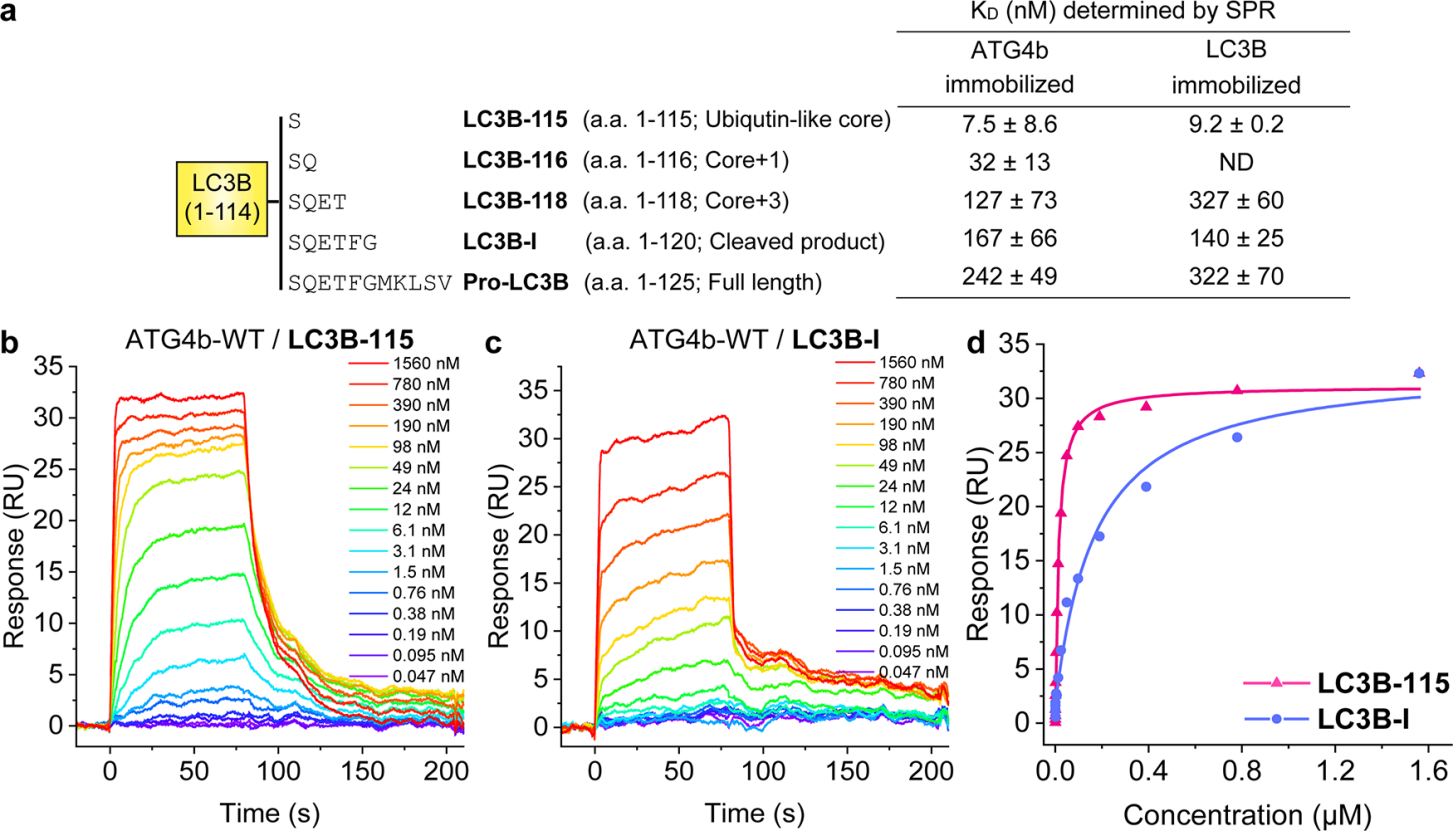
(a) LC3B truncations and their binding affinities to ATG4b-WT by surface plasmon resonance (SPR). Not determined (ND). (b, c) Representative sensorgrams and equilibrium binding models are shown for ATG4b binding to (b) immobilized biotin-LC3B-115, *K*_D_ = 9.2 nM and (c) immobilized biotin-LC3B-I, *K*_D_ = 140 nM. Doses range from 0.05 nM–1.6 μM ATG4b. (d) Binding isotherms for data from (b, c). Data are representative of three independent experiments.

Human pro-LC3B contains 125 residues comprised of a ubiquitin-like core and a C-terminal tail. ATG4b cleaves the last 5 residues of pro-LC3B to expose Gly120 for further lipidation. In the co-structure of LC3B bound to ATG4b-354 (a construct lacking residues 355–393), the C-terminal tail of LC3B occupies the substrate groove of ATG4b, while its ubiquitin-like core interacts with the surface of ATG4b and buries 1679 Å^2^ of the surface area.^14^ Gln116, Phe119, and Gly120 of LC3B each make substantial interactions with ATG4b.^14^ To further characterize the PPI between LC3B and ATG4b, we made the following truncations in LC3B: Ser115 (residues 1–115) to represent the ubiquitin-like core, Gln116 (residues 1–116, core+1 residue), Thr118 (residues 1–118, core+3 residues), and Gly120 (residues 1–120, LC3B-I) (Figure 1a). Hereafter, we refer to these constructs as LC3B-115, LC3B-116, LC3b-118, and LC3B-I, respectively. We also generated each LC3B truncation and pro-LC3B (residues 1–125) with N-terminal AviTags for *in vivo* biotinylation in *Escherichia coli*. All truncations were expressed in *E. coli* and purified similarly to the pro-LC3B (see the Materials and Methods Section); we found the LC3B truncations did not affect their expression levels (∼1 mg L^–1^ of culture). The identity and purity of proteins were confirmed by sodium dodecyl sulfate–polyacrylamide gel electrophoresis (SDS–PAGE) and mass spectrometry (Figure S1 and Table S1).

### Truncating the C-Terminal Tail of LC3B Enhanced the Binding Affinity for ATG4b

We measured LC3B proteins binding to ATG4b by SPR, using biotinylated LC3B immobilized on neutravidin-coated SPR sensor chips. Pro-LC3B was bound to full-length ATG4b with a *K*_D_ = 321 nM, a somewhat higher affinity than the reported *K*_M_ value of 5 μM (Figure 1a).^16,29^ LC3B-I bound to ATG4b with ∼2-fold higher affinity (*K*_D_ = 140 nM), and further truncation to LC3B-118 yielded a dissociation constant similar to the substrate (*K*_D_ = 326 nM). Surprisingly, LC3B-115 demonstrated the tightest binding, with *K*_D_ = 9.2 nM, 15-fold tighter than LC3B-I (Figure 1a–c). To confirm that immobilization of the LC3B proteins did not alter their binding potential or conformational flexibility, we also assessed binding by immobilizing biotinylated, full-length ATG4b on the sensor chip. We obtained similar *K*_D_ values in this format and confirmed a *K*_D_ ∼9 nM for LC3B-115 (Figure 1a). To validate that the tight binding of LC3B-115 was specific for ATG4b, we also added ATG4a to immobilized LC3B-115. ATG4a shares ∼55% identity with ATG4b but does not cleave LC3B.^16,30^ As expected, our SPR data showed that ATG4a did not bind to LC3B-115 (data not shown). The binding kinetics of LC3B-I vs. LC3B-115 binding to ATG4b largely conformed to expectations, in that tight-binding LC3B-115 showed faster association than the lower affinity LC3B-I (Table S2). Based on the published structures of ATG4b-354 bound to LC3B-I and pro-LC3B,^24^ the active site of ATG4b undergoes a conformational change to accommodate the C-terminal tail of LC3B. Overall, the conformational change may cause a decrease in the free energy of LC3B-I binding to ATG4b when compared to LC3B-115.

We next sought to establish the binding between LC3B-115 and full-length ATG4b using an orthogonal approach that did not require immobilization. Isothermal calorimetry (ITC) directly measured the heat released during binding, allowing us to determine the free energy/*K*_D_ and stoichiometry (*N*). With this method, the *K*_D_ values for LC3B-115 and LC3B-I binding to ATG4b were determined to be 143 and 4700 nM, respectively (Figure S2). The binding affinities calculated from ITC experiments were weaker than those determined by SPR; nevertheless, ITC-determined *K*_D_ values show a 30-fold difference between the binding of LC3B-I and LC3B-115, similar to the 15-fold difference observed by SPR (Figure 1a–c). Binding stoichiometry was calculated to be ∼1:1 (0.9 ± 0.2).

### LC3B-115 Inhibited ATG4b Hydrolysis of Pro-LC3B, but Had no Effect on the Small Peptide Substrate-33

**Figure 2:**
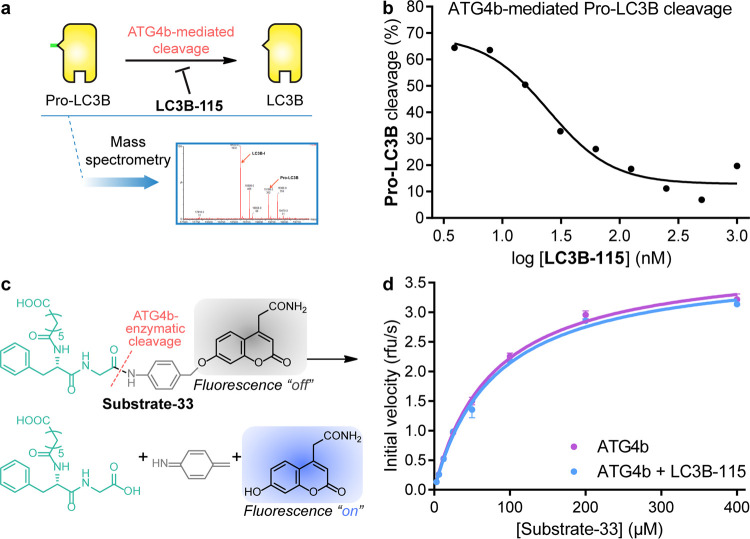
Effect of LC3B-115 on ATG4b activity. (a) Schematic illustration of the mass spectrometry-based assay to monitor the cleavage of pro-LC3B to LC3B-I in the presence of LC3B-115. (b) The cleavage of 1 μM pro-LC3B by 50 nM ATG4b in the presence of increasing concentrations of LC3B-115 was monitored by mass spectrometry. LC3B-115 inhibited the cleavage with an IC_50_ ∼15 nM. (c) Scheme of the ATG4b-mediated cleavage of peptide substrate-33. (d) Steady-state kinetic measurement of ATG4b-mediated cleavage of the fluorescent peptide substrate-33 in the presence and absence of 50 μM LC3B-115.

On the basis of these observations, we set out to demonstrate whether LC3B-115 acted as an inhibitor of ATG4b. We designed a mass spectrometry-based assay to monitor the cleavage of pro-LC3B to LC3B-I (Figure S3) and determined that LC3B-115 inhibited ATG4b cleavage of pro-LC3B with an IC_50_ = 15 nM (Figure 2a), in agreement with the *K*_D_ determined by SPR (Figure 1a–c). This study suggested that LC3B-115 competed for binding to ATG4b with pro-LC3B, likely through binding to the same binding site on ATG4b.

**Figure 3:**
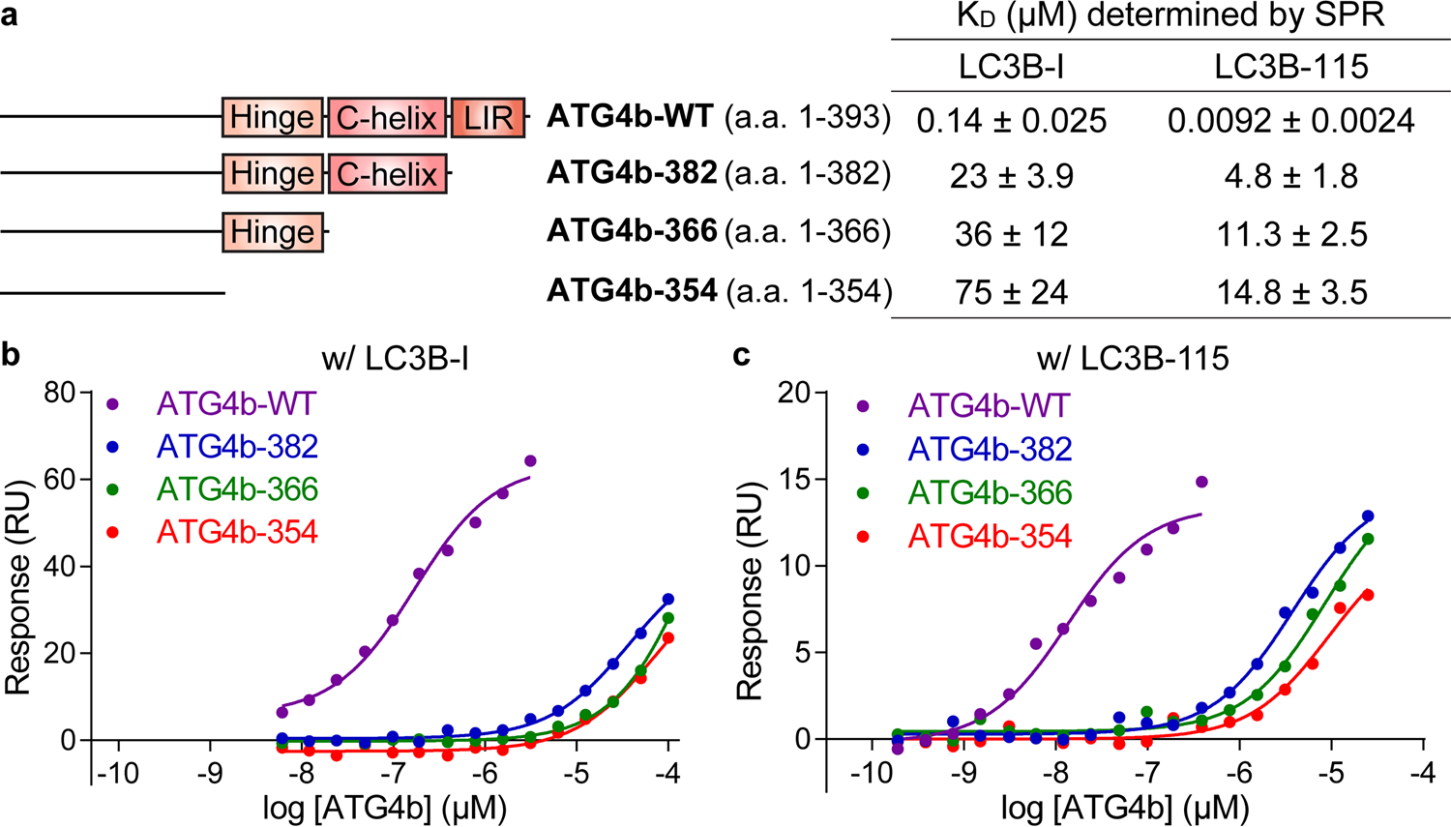
Evaluation of binding between LC3B and ATG4b truncations by SPR. (a) ATG4b truncations and their binding affinities to LC3B-I and LC3B-115. (b) Equilibrium binding curves for immobilized biotin-LC3B-I binding to ATG4b-WT, ATG4b-382, ATG4b-366, and ATG4b-354. (c) Equilibrium binding curves for immobilized biotin-LC3B-115 binding to ATG4b-WT, ATG4b-382, ATG4b-366, and ATG4b-354.

ATG4b is thought to exist in a low-activity state until LC3B binds and stabilizes the active site in a catalytically competent conformation.^14,25^ We evaluated whether LC3B-115 binding to ATG4b was able to induce the active conformation by measuring the catalytic efficiency of cleaving a small peptide analogue called substrate-33 (Figure 2b)^22^ in the absence or presence of LC3B-115. We ran the assay similarly to the original report,^22^ but at higher concentrations of ATG4b (5 μM vs 400 nM), and obtained the same Michaelis constant (*K*_M_ ∼50 μM).^22^ As shown in Figure 3c, the presence of LC3B-115 had no effect on the hydrolysis of the peptide substrate-33, implying that the binding of the LC3B-115 ubiquitin-like core did not induce the active conformation of ATG4b.

### Design of ATG4b Truncation Constructs

We then investigated the PPI from the ATG4b side. Rasmussen et al. have described a canonical LC3B interacting region (LIR) in the C-terminal region of ATG4b.^27^ LIR is characterized by [W/F/Y]XX[L/I/V] and typically preceded by several acidic residues; the two large hydrophobic residues, called HP1 and HP2, are critical in binding LC3B. Several LIR peptides have been crystallized with LC3B or its homologues, and it was found that LIR peptides could interact with LC3B in two opposite orientations while maintaining the hydrophobic interactions with HP1 and HP2 sites on LC3B.^31^ To dissect contributions from the C-terminal tail of ATG4b to the ATG4b/LC3B PPI, we used the sequence of human ATG4b as a query and ran a BLAST search in the protein data bank to design C-terminal truncation constructs based on potential structural motifs. As shown in the sequence alignment (Figure S4a), the last 11 residues (383–393) were homologous to the LIR motif derived from the selective autophagy receptor p62 that has been co-crystallized with LC3B (PDB: 2ZJD(32)). We also found that the region containing residues 363–382 was homologous to a helical region in the ATG7-LC3B co-structure (PDB: 3RUI, Figure S4b). In the apo structure of ATG4b (PDB: 2CY7), residues 371–376 formed a C-terminal helix, indicating that this region of ATG4b had an intrinsic helical propensity.^25^ Guided by these results, we made three C-terminal truncations at Asp382 (aa 1–382, deletion of the LIR region), Asn366 (aa 1–366, deletion of both LIR and helix-homology regions), and Leu354 (aa 1–354, the construct used for ATG4b-LC3B co-crystallography) (Figure 3a). Hereafter, we refer to these truncation constructs as ATG4b-382, ATG4b-366, and ATG4b-354. Like LC3B truncations, ATG4b truncations did not affect the expression levels (50 mg L^–1^ of *E. coli* culture; Figure S5).

### C-Terminal Truncations of ATG4b Abolished High-Affinity Binding to LC3B

We assessed binding between ATG4b truncations and LC3B using SPR. N-Terminally biotinylated LC3B was immobilized on a neutravidin-coated SPR surface and ATG4b proteins (WT, ATG4b-382, ATG4b-366, and ATG4b-354) were flowed over the surface to determine binding affinity and binding kinetics. ATG4b-382 showed a 160- and 500-fold loss of binding to LC3B-I and LC3B-115, respectively (Figures 3b,c, S6, S7, and Table S2). Further truncation of the helical region and residues 355–365 led to an additional 3-fold loss of binding affinity for both LC3B constructs (Figure 3a). These data verified that the LIR region at the C-terminal tail of ATG4b played a critical role in binding to the ubiquitin-like core of LC3B.

### Hydrolysis of Pro-LC3B, but not of Peptide Substrate-33, Was Sensitive to C-Terminal Truncations of ATG4b

**Figure 4:**
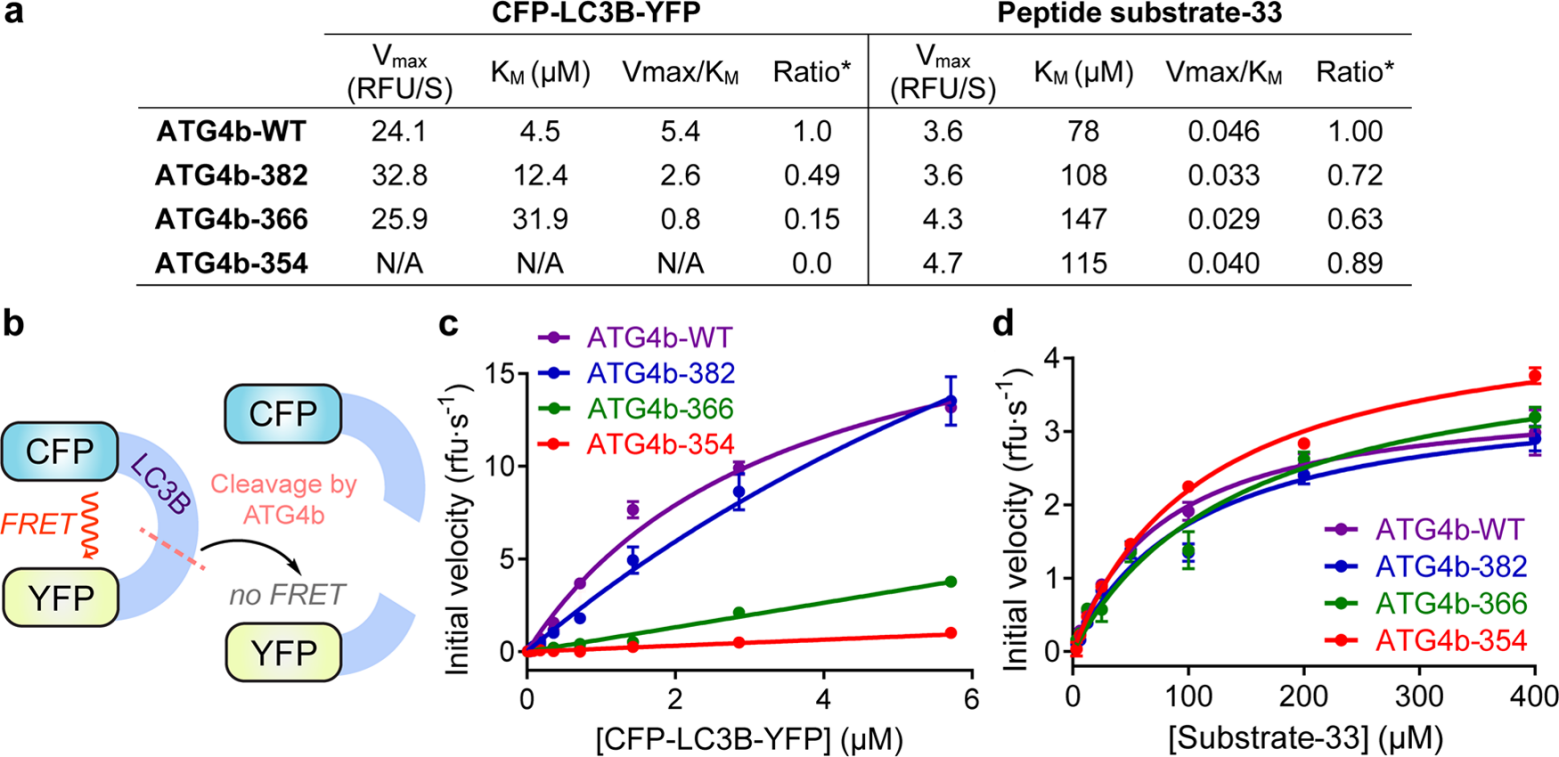
Steady-state kinetic analysis of ATG4b constructs. (a) Kinetic parameters for the cleavage of the FRET-LC3B substrate (CFP-LC3B-YFP) and peptide substrate-33 by truncation constructs of ATG4b. (b) Schematic illustration of the FRET assay using the CFP-LC3B-YFP substrate. (c) Turnover of substrate CFP-LC3B-YFP by ATG4b-WT, ATG4b-382, ATG4b-366, and ATG4b-354. (d) Turnover of peptide substrate-33 by ATG4b-WT, ATG4b-382, ATG4b-366, and ATG4b-354. The linear portions of initial rates of product formation were used to determine the kinetic parameters.

Given the effects of the C-terminal tail of ATG4b on LC3B binding, we expected to see the loss of catalytic efficiency of C-terminal ATG4b truncations in hydrolyzing pro-LC3B to LC3B-I. To test the cleavage of full-length pro-LC3B, we developed a Förster Resonance Energy Transfer (FRET)-based cleavage assay. We adopted a fusion protein^29^ in which cyan fluorescent protein (CFP) was fused to the N-terminus of pro-LC3B, while yellow fluorescent protein (YFP) was fused to the C-terminus. Based on the rates of proteolysis of CFP-proLC3B-YFP (Figure 4a,b),^29^ ATG4b-382 lost 50% of activity based on *V*_max_/*K*_M_; ATG4b-366 lost 85% activity, while ATG4b-354 showed essentially no activity toward the FRET substrate (Figure 4c).

The loss of activity was due to weakening of LC3B binding as judged by *K*_M_ (Figure 4a). Deletion of the LIR in the C-terminus of ATG4b (ATG4b-382) had little effect on its catalytic turnover rate but led to a 2.8-fold reduction in LC3B-substrate binding as estimated by *K*_M_. Further truncation of the C-helix did not affect the turnover rate but further reduced the substrate binding by 2.6-fold. Although C-terminal truncations ATG4b-366 and ATG4b-354 showed little activity, as we expected, ATG4b-382 showed surprisingly more activity than the binding data suggested. Interestingly, a similar phenomenon was observed and reported in cellular assays,^27^ as similar truncation constructs lost up to 90% of binding to LC3B in pull-down assays, but retained activity in generating LC3B-I.

We then evaluated the ability of the ATG4b C-terminal truncations to hydrolyze peptide substrate-33 (Figure 4d). In contrast to the effects on hydrolysis of full-length pro-LC3B, the C-terminal truncations of ATG4b did not significantly affect the hydrolysis of substrate-33, indicating that the C-terminal tail of ATG4b was not involved in substrate recognition or turnover at the active site.

## Discussion

LC3B is a key protein in mediating autophagosome formation and is used as a biomarker in studying autophagy. Here, we dissect the PPI between LC3B and its protease ATG4b and describe the interactions between ATG4b and the LC3B core domain and C-terminal tail. Using biochemical and molecular biology methods, we designed different LC3B deletion proteins to dissect the contributions from its ubiquitin-like core (LC3B-115) and its C-terminal tail. We discovered that LC3B-115 has an affinity ∼15–30-fold tighter than LC3B-I or pro-LC3B. Consistent with the binding data, LC3B-115 potently inhibits ATG4b proteolysis of pro-LC3B to LC3B-I with an IC_50_ = 15 nM. This IC_50_ might be an underestimate since 50 nM ATG4b was used in the assay; nevertheless, inhibition closely matches the calculated *K*_d_ from SPR (9 nM).

Similarly, we also evaluated the contribution of ATG4b, particularly its C-terminal tail, to the PPI with LC3B. Recent studies highlighted the existence of a canonical LIR in the C-terminal tail of ATG4b, which contributed significantly to its binding to LC3B.^27^ Here, we provided quantitative analysis of the role of this C-terminal tail in forming the PPI. Based on sequence alignments, we divided the C-terminal 39 residues of ATG4b into three structural motifs: (1) the LIR (residues 383–393), (2) the C-helix (residues 367–382), and (3) a hinge region (residues 355–366). We then truncated ATG4b at each motif to evaluate its effect on LC3B binding. We show that the loss of the LIR motif dramatically decreases the binding affinity to LC3B by ∼200–500-fold. Further truncation of the C-helix and the “hinge” decreases the binding affinity by an additional ∼2–3-fold. Interestingly, our quantitative kinetic data show for the first time that these 39 residues at the C-terminus of ATG4b do not affect the rate of turnover (*V*_max_) of either pro-LC3B or the peptide substrate-33, but they do significantly contribute to LC3B binding as shown by the values of *K*_D_ from SPR and ITC and *K*_M_ from the enzymatic assay. These data thus verify that the LIR region at the C-terminal tail of ATG4b plays a critical role in binding the ubiquitin-like core of LC3B.

**Figure 5:**
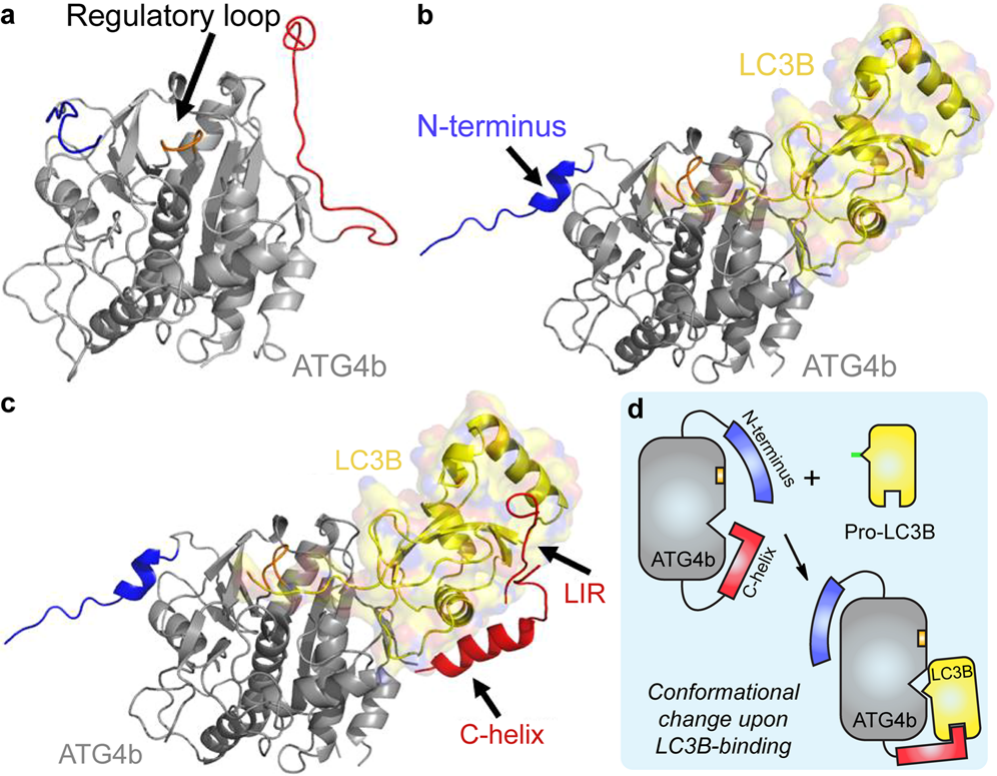
Structural model for the LC3B-ATG4b interaction. (a) X-ray structure of apo-ATG4b (2CY7). (b) X-ray crystal structure of pro-LC3B/ATG4b-354 (2ZZP). The main body of ATG4b is shown in gray and LC3B is shown in yellow. Upon LC3B binding, the N-terminal tail (blue) and the regulatory loop (orange) of ATG4b undergo conformational changes. The C-terminal tail (red) is deleted in the complex structure. (c) Model of full-length ATG4b-LC3B. The model is built from 2CY7, 2ZZP, the ATG7c/LC3 complex (3RUI), and the LC3/p62 peptide complex “LIR” (2ZJD). We hypothesize that the C-terminus (residues 355–393) of ATG4b binds to the back of LC3, perhaps forming a helix, as is seen in the complex of ATG7c bound to LC3. Images were made using Pymol. (d) Schematic illustration of the binding modes described in (a∼c).

Although the X-ray crystal structures for ATG4b-354/LC3B and ATG4b LIR/GABARAPL1 complexes are available, there is no crystal structure of full-length ATG4b bound to LC3B; therefore, a pictorial view on how the C-terminal 39 residues of ATG4b contribute to LC3B binding is lacking. Our data points to a bipartite interaction of LC3B with ATG4b, in which binding of the C-terminal tail of pro-LC3B and reorganization of the active site of ATG4b are separable from the binding of the LC3B-115 core to the C-terminal tail of ATG4b (Figure 5). Binding of LC3B-115 has no effect on the activity of the peptide substrate-33, suggesting that the two binding events are not allosterically coupled. The lower affinity of pro-LC3B and LC3B-I compared to LC3B-115 is also consistent with the notion that energy is required to change the conformation of the ATG4b active site. On the C-terminal side of ATG4b, truncations have no effect on *k*_cat_, but give a weaker *K*_M_ for pro-LC3B; by contrast, the C-terminal truncations have no effect on *k*_cat_ or *K*_M_ when peptide substrate-33 serves as the substrate.

In the apo structure of ATG4b, the C-terminal tail occupies the site where the LC3B core binds to the LC3B/ATG4b-354 structure. Hence, the C-terminal tail of ATG4b must adopt a different conformation in the LC3B-bound complex. The truncation data are consistent with the C-terminal tail wrapping around the LC3B core domain to confer a significant portion of the binding energy. The quantitative enzymology and binding data are consistent with the estimated 75–90% loss in the ability of the ATG4b-382 mutant to bind LC3B in lysates.^27^ Taken together, our findings shed new light on the PPI between LC3B and ATG4b. In particular, LC3B-115 could serve as a potent inhibitor of ATG4b, suitable as a tool for cell-based studies of the ATG4b function, and could inspire peptidomimetic or small-molecule design of novel drugs for inhibiting autophagy.

## Materials and Methods

### Cloning, Expression, and Purification of ATG4b Proteins

The plasmid for ATG4b-WT was provided by the Sanford Burnham Institute. The plasmids encoding ATG4b truncation proteins were cloned based on the WT plasmid through standard cloning. The primers used for cloning are listed in Tables S3 and S4. Inserts in all plasmids were confirmed by sequencing. The plasmids were transformed into the *E. coli* Rosetta 2 (DE3) strain (Invitrogen). Frozen cell stocks in 25% glycerol were streaked onto an ampicillin (200 g mL^–1^) plate and grown overnight. One colony was picked and grown in a starter culture and used to inoculate 6 L of 2X YT media. Upon log-phase growth (OD_600_ ∼0.6–0.8), expression was carried out by overnight induction with 0.2 mM IPTG at 16 °C. The cells were harvested at 5000 rpm for 15 min and resuspended in 100 mM NaCl, 100 mM Tris pH 8.0 and disrupted through a microfluidizer and the lysate was then spun down at 20,000 rpm for 45 min and filtered. The protein was purified in two steps by Ni-NTA affinity chromatography and anion exchange chromatography using an ÄKTA system (GE Healthcare). The lysate was then loaded onto a 1 mL HisTrap HP column (GE Healthcare). The column was subsequently washed with 10% buffer B (100 mM Tris pH 8.0, 100 mM NaCl, 200 mM imidazole, pH 8.0) and 20% B, and eluted with 100% B. The Ni elution fraction was diluted 10-fold with 20 mM Tris pH 8.0 and was loaded onto a 1 mL HiTrap Q column (GE Healthcare). Elution was carried out by a 0–100% 1 M NaCl gradient over 20 column volumes collecting 1.0 mL fractions. Flow rates were typically held constant at 1.0 mL min^–1^ or lowered if the pressure exceeded the limit of the column accordingly. Proteins were concentrated in Amicon centrifugal filters and stored in a −80 °C freezer. Protein concentrations were determined by A280 using a Nanodrop (Thermo). The extinction coefficient at A280 (in mg/mL) was calculated based on the amino acid sequence using ExPASy (https://web.expasy.org/protparam/).

### Cloning, Expression, and Purification of LC3B

The cDNA fragment of LC3B was purchased from Origene. The full-length LC3B and truncated proteins were cloned into the pET15b vector. The primers are listed in Tables S5 and S6. All LC3B proteins were expressed and purified similarly to the ATG4b proteins. Protein concentrations were determined by A280 using a NanoDrop Spectrophotometer (Thermo). The extinction coefficient at A280 (in mg mL^–1^) was calculated based on the amino acid sequence using ExPASy (https://web.expasy.org/protparam/).

### Cloning, Expression, and Purification of Biotinylated Proteins

The DNA encoding ATG4b or LC3B was cloned into the pET15b vector with an N-terminal His6-Tev-Avi tag. The plasmid was co-transformed with a second plasmid encoding BirA (kanamycin resistance) into the *E. coli* Rosetta BL21 strain (Invitrogen). Cells were grown to OD_600nm_ ∼0.6–0.8, 50 μM biotin in 10 mM bicine buffer (pH 8.3) was added to media followed by overnight induction with 0.2 mM IPTG at 16 °C. Cells were harvested, proteins were purified, and concentrations quantified as described above.

### ITC Experiment

All titrations were carried out at 25 °C in a MicroCal T200 isothermal titration calorimeter (Malvern). Proteins were dialyzed into 20 mM HEPES (pH 7.5), 200 mM NaCl, and 0.5 mM Tris(2-carboxyethyl)phosphine (TCEP) overnight. Concentrations of proteins were varied from run-to-run to balance signal intensity (high concentrations) and accuracy for measuring low *K*_D_ values (low concentrations); 10–68 μM LC3B proteins were loaded in the cell and a 10-fold higher concentration of ATG4b proteins was loaded in the syringe. Heat of dilution was subtracted and data were fitted to a single-site binding model using Origin software and models provided with the instrument (MicroCal).

### SPR Experiment

All SPR experiments were carried out using a Biacore 4000 instrument (GE healthcare). NeutrAvidin-coated sensor chips were prepared on CM5 chips as follows: the surface was activated by injecting a 1:1 mixture of 60 mM *N*-hydroxysuccinimide and 240 mM 1-ethyl-3-(3-dimethylaminopropyl)-carbodiimide for 7 min, followed by a 7 min injection of 0.25 mg mL^–1^ NeutrAvidin (Thermo Scientific) in 10 mM acetic acid (pH 4.5). The surface was then blocked by a 2-min injection of 1 M ethanolamine (pH 8.3).

Purified biotinylated ATG4b or LC3B constructs were immobilized in 1× PBS pH 7.4, 0.5 mM TCEP, and 0.05% Tween 20. Protein was immobilized to 10–80 resonance units by injecting 62–500 ng mL^–1^ of protein for 1 min at 25 °C. Protein immobilization was followed by a 2 min injection of 0.2 mg mL^–1^ amino-polyethylene glycol-biotin (Thermo Scientific) to block any remaining biotin binding sites. A reference surface was created using the same protocol with the omission of the protein injection step. Binding between ATG4b and LC3B was measured in SPR running buffer 1× PBS, 0.5 mM TCEP, and 0.05% Tween 20 at 20 °C. Then, 0.2 nM–20 μM proteins were injected at a flow rate of 30 μL mL^–1^ for 90–180 s, with a dissociation time of 180–600 s. Data were double-reference subtracted (references include analyte flowed over a biotin-only surface and buffer only flowed over the immobilized protein sample) and fitted to a 1:1 binding model using the Biacore 4000 evaluation software provided with the instrument.

### Substrate-33 Assay

Substrate 33 was generously provided by Prof. Robert N. Young (Simon Fraser University, Canada) and was dissolved in DMSO at a stock concentration of 25 mM. The peptide substrate was then further diluted in assay buffer (50 mM HEPES pH 7.5, 150 mM NaCl, 500 μM TCEP) to various concentrations and incubated with 50 μM ATG4b proteins. Cleavage of substrate-33 was monitored on Flexstation III (Molecular device) continuously for 2 h. The linear portion of initial rates was used to plot the Michaelis–Menten graph to generate the kinetic parameters.

### CFP-LC3B-YFP Assay

CFP-LC3B-YFP was produced as previously reported. Due to the overlap emission spectrum between CFP and YFP, we followed the paper by Liu et al,^33^ and ran emission and excitation scans for separated CFP-LC3B and YFP on a FlexStation III plate reader (Molecular Device) and used the formula in the paper to calculate the neat FRET signal. Various concentrations of CFP-LC3B-YFP were incubated with 50 nM ATG4b proteins in the assay buffer (50 mM HEPES pH 7.5, 150 mM NaCl, 500 μM TCEP), the rate of cleavage was monitored on a FlexStation III (Molecular Device).

### Mass-Based LC3B Cleavage Assay

Various concentrations of LC3B-115 were added to a reaction mixture containing 50 nM ATG4b and 1 μM pro-LC3B in the assay buffer, the reaction was quenched after 5 min by loading onto a Xevo Mass Spectrometer (Waters Corporation). The percentage of cleavage was calculated based on the mass intensity for pro-LC3B and LC3B-I.

## Supporting Information Available

Evaluation of binding between LC3B truncations and ATG4b by isothermal calorimetry (ITC); sequence alignment of the C-terminal tail of ATG4b with (a) p62 tail or (b) ATG7; representative SPR sensorgrams of ATG4b proteins binding to LC3B-I fitted to a 1:1 kinetic binding model; expected masses and observed masses for LC3B constructs; primers used to make ATG4b constructs; primers used to make LC3B constructs, and Supporting sequences (PDF)

## Accession Codes

ATG4b (UniProt Accession ID: Q9Y4P1) and LC3B (UniProt Accession ID: Q9GZQ8)

## References

[ref1] Drag M.; Salvesen G. S. Emerging principles in protease-based drug discovery. Nat. Rev. Drug Discovery 2010, 9, 690–701. 10.1038/nrd3053. 20811381 PMC2974563

[ref2] López-Otín C.; Overall C. M. Protease degradomics: A new challenge for proteomics. Nat. Rev. Mol. Cell Biol. 2002, 3, 509–519. 10.1038/nrm858. 12094217

[ref3] Fuentes-Prior P.; Iwanaga Y.; Huber R.; Pagila R.; Rumennik G.; Seto M.; Morser J.; Light D. R.; Bode W. Structural basis for the anticoagulant activity of the thrombin–thrombomodulin complex. Nature 2000, 404, 518–525. 10.1038/35006683. 10761923

[ref4] HUNTINGTON J. A. Molecular recognition mechanisms of thrombin. J. Thromb. Haemostasis 2005, 3, 1861–1872. 10.1111/j.1538-7836.2005.01363.x. 16102053

[ref5] Zhou Y. Y.; Wang Z. K.; Huang Y. J.; Bai C. J.; Zhang X. L.; Fang M. D.; Ju Z. Y.; Liu B. Membrane dynamics of ATG4B and LC3 in autophagosome formation. J. Mol. Cell Biol. 2022, 13, 853–863. 10.1093/jmcb/mjab059. 34562084 PMC8800521

[ref6] Abreu S.; Kriegenburg F.; Gómez-Sánchez R.; Mari M.; Sánchez-Wandelmer J.; Skytte Rasmussen M.; Soares Guimarães R.; Zens B.; Schuschnig M.; Hardenberg R.; Peter M.; Johansen T.; Kraft C.; Martens S.; Reggiori F. Conserved Atg8 recognition sites mediate Atg4 association with autophagosomal membranes and Atg8 deconjugation. EMBO Rep. 2017, 18, 765–780. 10.15252/embr.201643146. 28330855 PMC5412903

[ref7] Mizushima N. Autophagy: process and function. Genes Dev. 2007, 21, 2861–2873. 10.1101/gad.1599207. 18006683

[ref8] Mizushima N.; Klionsky D. J. Protein turnover via autophagy: Implications for metabolism. Annu. Rev. Nutr. 2007, 27, 19–40. 10.1146/annurev.nutr.27.061406.093749. 17311494

[ref9] Yang Z. F.; Klionsky D. J. Eaten alive: a history of macroautophagy. Nat. Cell Biol. 2010, 12, 814–822. 10.1038/ncb0910-814. 20811353 PMC3616322

[ref10] Levine B.; Klionsky D. J. Development by self-digestion: Molecular mechanisms and biological functions of autophagy. Dev. Cell 2004, 6, 463–477. 10.1016/S1534-5807(04)00099-1. 15068787

[ref11] Tanida I.; Sou Y. S.; Ezaki J.; Minematsu-Ikeguchi N.; Ueno T.; Kominami E. HsAtg4B/HsApg4B/autophagin-1 cleaves the carboxyl termini of three human Atg8 homologues and delipidates microtubule-associated protein light chain 3-and GABA(A) receptor-associated protein-phospholipid conjugates. J. Biol. Chem. 2004, 279, 36268–36276. 10.1074/jbc.M401461200. 15187094

[ref12] Kirisako T.; Ichimura Y.; Okada H.; Kabeya Y.; Mizushima N.; Yoshimori T.; Ohsumi M.; Takao T.; Noda T.; Ohsumi Y. The reversible modification regulates the membrane-binding state of Apg8/Aut7 essential for autophagy and the cytoplasm to vacuole targeting pathway. J. Cell Biol. 2000, 151, 263–275. 10.1083/jcb.151.2.263. 11038174 PMC2192639

[ref13] Ichimura Y.; Kirisako T.; Takao T.; Satomi Y.; Shimonishi Y.; Ishihara N.; Mizushima N.; Tanida I.; Kominami E.; Ohsumi M.; Noda T.; Ohsumi Y. A ubiquitin-like system mediates protein lipidation. Nature 2000, 408, 488–492. 10.1038/35044114. 11100732

[ref14] Satoo K.; Noda N. N.; Kumeta H.; Fujioka Y.; Mizushima N.; Ohsumi Y.; Inagaki F. The structure of Atg4B-LC3 complex reveals the mechanism of LC3 processing and delipidation during autophagy. EMBO J. 2009, 28, 1341–1350. 10.1038/emboj.2009.80. 19322194 PMC2683054

[ref15] Fujita N.; Hayashi-Nishino M.; Fukumoto H.; Omori H.; Yamamoto A.; Noda T.; Yoshimori T. An Atg4B mutant hampers the lipidation of LC3 paralogues and causes defects in autophagosome closure. Mol. Biol. Cell 2008, 19, 4651–4659. 10.1091/mbc.e08-03-0312. 18768752 PMC2575160

[ref16] Li M.; Hou Y.; Wang J.; Chen X.; Shao Z. M.; Yin X. M. Kinetics comparisons of mammalian Atg4 homologues indicate selective preferences toward diverse Atg8 substrates. J. Biol. Chem. 2011, 286, 7327–7338. 10.1074/jbc.M110.199059. 21177865 PMC3044989

[ref17] Kauffman K. J.; Yu S. L.; Jin J. X.; Mugo B.; Nguyen N.; O’Brien A.; Nag S.; Lystad A. H.; Melia T. J. Delipidation of mammalian Atg8-family proteins by each of the four ATG4 proteases. Autophagy 2018, 14, 992–1010. 10.1080/15548627.2018.1437341. 29458288 PMC6103404

[ref18] Agrotis A.; Pengo N.; Burden J. J.; Ketteler R. Redundancy of human ATG4 protease isoforms in autophagy and LC3/GABARAP processing revealed in cells. Autophagy 2019, 15, 976–997. 10.1080/15548627.2019.1569925. 30661429 PMC6526816

[ref19] Mariño G.; Fernandez A. F.; Cabrera S.; Lundberg Y. W.; Cabanillas R.; Rodriguez F.; Salvador-Montoliu N.; Vega J. A.; Germana A.; Fueyo A.; Freije J. M. P.; Lopez-Otin C. Autophagy is essential for mouse sense of balance. J. Clin. Invest. 2010, 120, 2331–2344. 10.1172/JCI42601. 20577052 PMC2898610

[ref20] Amaravadi R. K.; Lippincott-Schwartz J.; Yin X. M.; Weiss W. A.; Takebe N.; Timmer W.; DiPaola R. S.; Lotze M. T.; White E. Principles and current strategies for targeting autophagy for cancer treatment. Clin. Cancer Res. 2011, 17, 654–666. 10.1158/1078-0432.CCR-10-2634. 21325294 PMC3075808

[ref21] Rebecca V. W.; Amaravadi R. K. Emerging strategies to effectively target autophagy in cancer. Oncogene 2016, 35, 1–11. 10.1038/onc.2015.99. 25893285 PMC4838040

[ref22] Vezenkov L.; Honson N. S.; Kumar N. S.; Bosc D.; Kovacic S.; Nguyen T. G.; Pfeifer T. A.; Young R. N. Development of fluorescent peptide substrates and assays for the key autophagy-initiating cysteine protease enzyme, ATG4B. Bioorg. Med. Chem. 2015, 23, 3237–3247. 10.1016/j.bmc.2015.04.064. 25979376

[ref23] Nguyen T. G.; Honson N. S.; Arns S.; Davis T. L.; Dhe-Paganon S.; Kovacic S.; Kumar N. S.; Pfeifer T. A.; Young R. N. Development of fluorescent substrates and assays for the key autophagy-related cysteine protease enzyme, ATG4B. Assay Drug Dev. Technol. 2014, 12, 176–189. 10.1089/adt.2013.561. 24735444 PMC3994995

[ref24] Maruyama T.; Noda N. N. Autophagy-regulating protease Atg4: structure, function, regulation and inhibition. J. Antibiot. 2018, 71, 72–78. 10.1038/ja.2017.104. PMC5799747 28901328

[ref25] Sugawara K.; Suzuki N. N.; Fujioka Y.; Mizushima N.; Ohsumi Y.; Inagaki F. Structural basis for the specificity and catalysis of human Atg4B responsible for mammalian autophagy. J. Biol. Chem. 2005, 280, 40058–40065. 10.1074/jbc.M509158200. 16183633

[ref26] Frey S.; Gorlich D. The *Xenopus laevis* Atg4B Protease: Insights into substrate recognition and application for tag removal from proteins expressed in pro- and eukaryotic hosts. PLoS One 2015, 10, e0125099 10.1371/journal.pone.0125099. 25923686 PMC4414272

[ref27] Skytte Rasmussen M.; Mouilleron S.; Shrestha B. K.; Wirth M.; Lee R.; Larsen K. B.; Princely Y. A.; O’Reilly N.; Sjottem E.; Tooze S. A.; Lamark T.; Johansen T. ATG4B contains a C-terminal LIR motif important for binding and efficient cleavage of mammalian orthologs of yeast Atg8. Autophagy 2017, 13, 834–853. 10.1080/15548627.2017.1287651. 28287329 PMC5446077

[ref28] Yang Z. F.; Wilkie-Grantham R. P.; Yanagi T.; Shu C. W.; Matsuzawa S.; Reed J. C. ATG4B (Autophagin-1) phosphorylation modulates autophagy. J. Biol. Chem. 2015, 290, 26549–26561. 10.1074/jbc.M115.658088. 26378241 PMC4646313

[ref29] Li M.; Chen X.; Ye Q. Z.; Vogt A.; Yin X. M. A high-throughput FRET-based assay for determination of Atg4 activity. Autophagy 2012, 8, 401–412. 10.4161/auto.18777. 22302004 PMC3337841

[ref30] Scherz-Shouval R.; Sagiv Y.; Shorer H.; Elazar Z. The COOH terminus of GATE-16, an intra-Golgi transport modulator, is cleaved by the human cysteine protease HsApg4A. J. Biol. Chem. 2003, 278, 14053–14058. 10.1074/jbc.M212108200. 12473658

[ref31] Birgisdottir Å. B.; Lamark T.; Johansen T. The LIR motif - crucial for selective autophagy. J. Cell Sci. 2013, 126, 3237–3247. 10.1242/jcs.126128. 23908376

[ref32] Hong S. B.; Kim B. W.; Lee K. E.; Kim S. W.; Jeon H.; Kim J.; Song H. K. Insights into noncanonical E1 enzyme activation from the structure of autophagic E1 Atg7 with Atg8. Nat. Struct. Mol. Biol. 2011, 18, 1323–1330. 10.1038/nsmb.2165. 22056771

[ref33] Liu Y.; Liao J. Y. Quantitative FRET (Forster resonance energy transfer) analysis for SENP1 protease kinetics determination. J. Visualized Exp. 2013, 72, e4430 10.3791/4430. PMC3605757 23463095

