# Supplementary material for: LC3B Binds to the Autophagy Protease ATG4b with High Affinity Using a Bipartite Interface
Source: Biochemistry. 2022 Oct 20;61(21):2295–302. doi: 10.1021/acs.biochem.2c00482 (PMC9631991; doi:10.1021/acs.biochem.2c00482)
Supplement: Supplementary file 1 — bi2c00482_si_001.pdf [file bi2c00482_si_001.pdf]

Supporting Information

LC3B binds to the autophagy protease ATG4b with high affinity using a bipartite interface

Yinyan Tang,[†,‡] *Amber Kay,*[†,‡] *Ziwen Jiang,*[†,‡] *Michelle R. Arkin*[*,†,‡]

[†]Department of Pharmaceutical Chemistry, [‡]Small Molecule Discovery Center, University of California, San Francisco, CA 94158, USA

[*]Email: michelle.arkin@ucsf.edu (M.R.A.)

Table of Contents

1. Supplementary figures and tables

Figure S1. (a) Representative mass spectra for LC3B constructs. The +178 peak is the a-N-gluconoylated version, a common post translational modification observed with proteins expressed with N-terminal His6 tag in *E. coli*. (b) SDS-PAGE protein gel of LC3B proteins.

Figure S2. Evaluation of binding between LC3B truncations and ATG4b by isothermal calorimetry (ITC).

Figure S3. A Representative mass spectrum for an MS-based assay to evaluate the cleavage of pro-LC3B.

Figure S4. Sequence alignment of the C-terminal tail of ATG4b with (a) p62 tail or (b) ATG7.

Figure S5. SDS-PAGE protein gel for all ATG4b constructs.

Figure S6. Representative SPR sensorgrams of ATG4b proteins binding to LC3B-I fit to a 1:1 kinetic binding model.

Figure S7. Representative SPR sensorgrams of ATG4b proteins binding to LC3B-115 fit to a 1:1 kinetic binding model.

Table S1. Expected masses and observed masses for LC3B constructs.

Table S2. Kinetic rate constants for binding of LC3B-I and LC3B-115 to ATG4b truncations.

Table S3. Primers used to make ATG4b constructs.

Table S4. Primer combinations for ATG4b pDuet vector constructs.

Table S5. Primers used to make LC3B constructs.

Table S6. Primer combinations for LC3B N-His-Avi vector constructs.

2. Supplementary sequences

1. Supplementary figures and tables

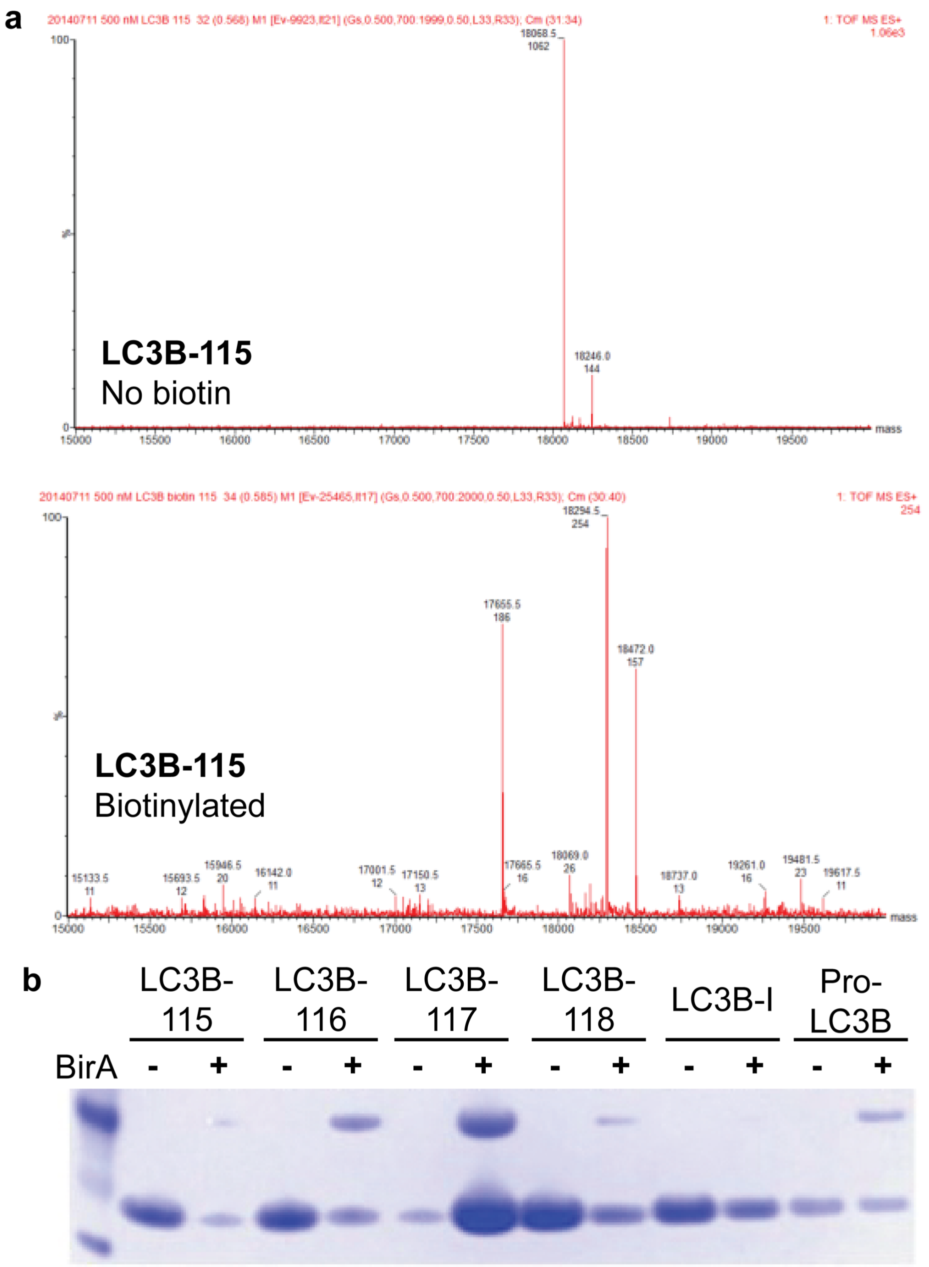

Figure S1. (a) Representative mass spectra for LC3B constructs. The +178 peak is the a-N-gluconoylated version, a common post translational modification observed with proteins expressed with N-terminal His6 tag in *E. coli*. (b) SDS-PAGE protein gel of LC3B proteins.

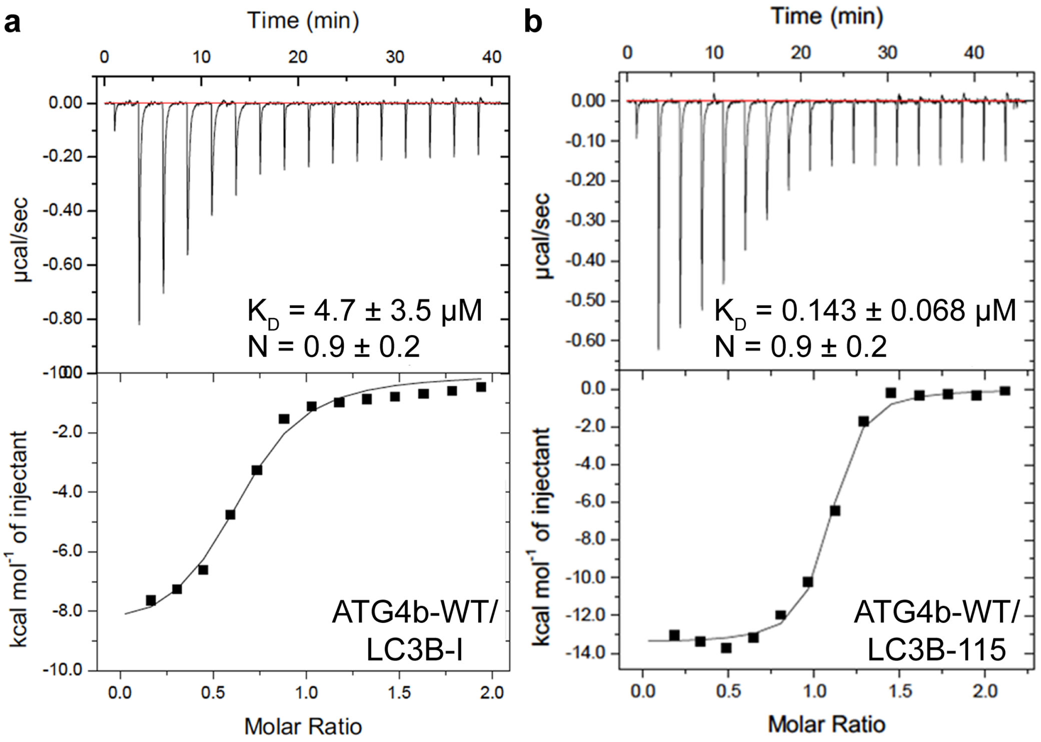

Figure S2. Evaluation of binding between LC3B truncations and ATG4b by isothermal calorimetry (ITC). Representative data are shown for ATG4b WT binding to LC3B-I and LC3B-115: (a) A 700-μM solution of ATG4b was titrated into 68 μM LC3B-I; (b) 100 μM solution of ATG4b titrated into 10 μM LC3B-115. N and K_D values are the mean and standard deviation for five independent experiments; data are shown for one representative measurement.

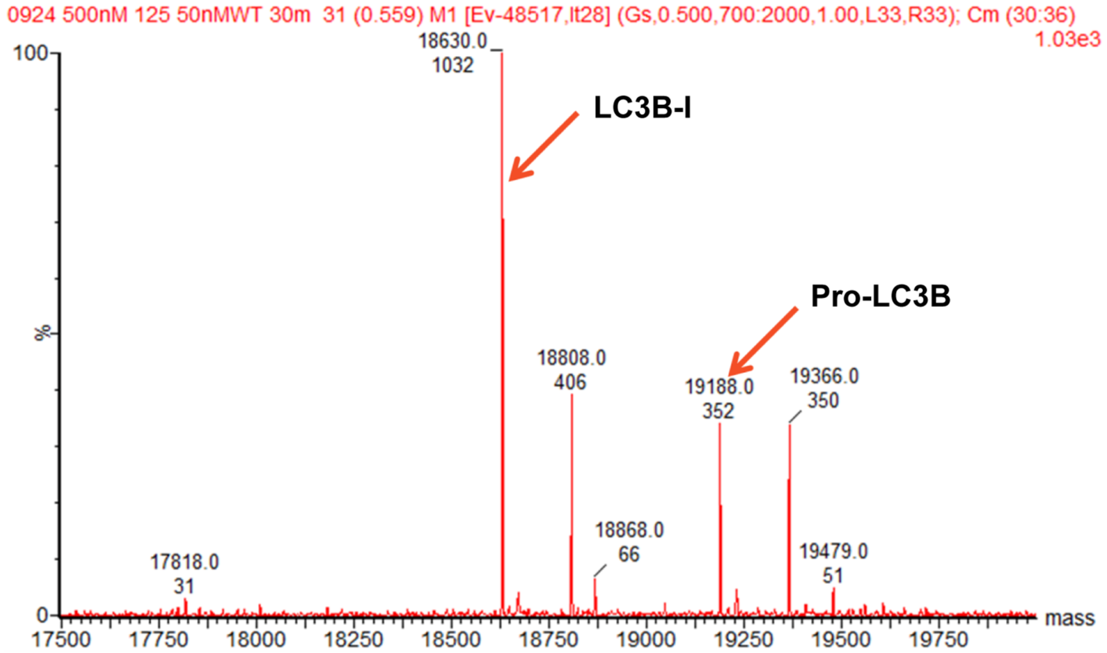

Figure S3. A Representative mass spectrum for an MS-based assay to evaluate the cleavage of pro-LC3B. The arrows are pointing to the masses corresponding to pro-LC3B (substrate) and LC3B-I (product), respectively.

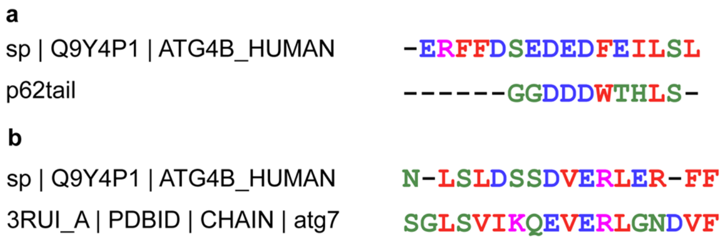

Figure S4. Sequence alignment of the C-terminal tail of ATG4b with (a) p62 tail or (b) ATG7.

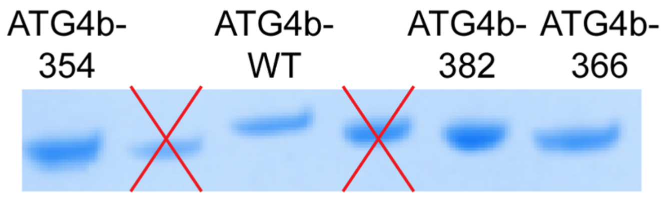

Figure S5. SDS-PAGE protein gel for all ATG4b constructs.

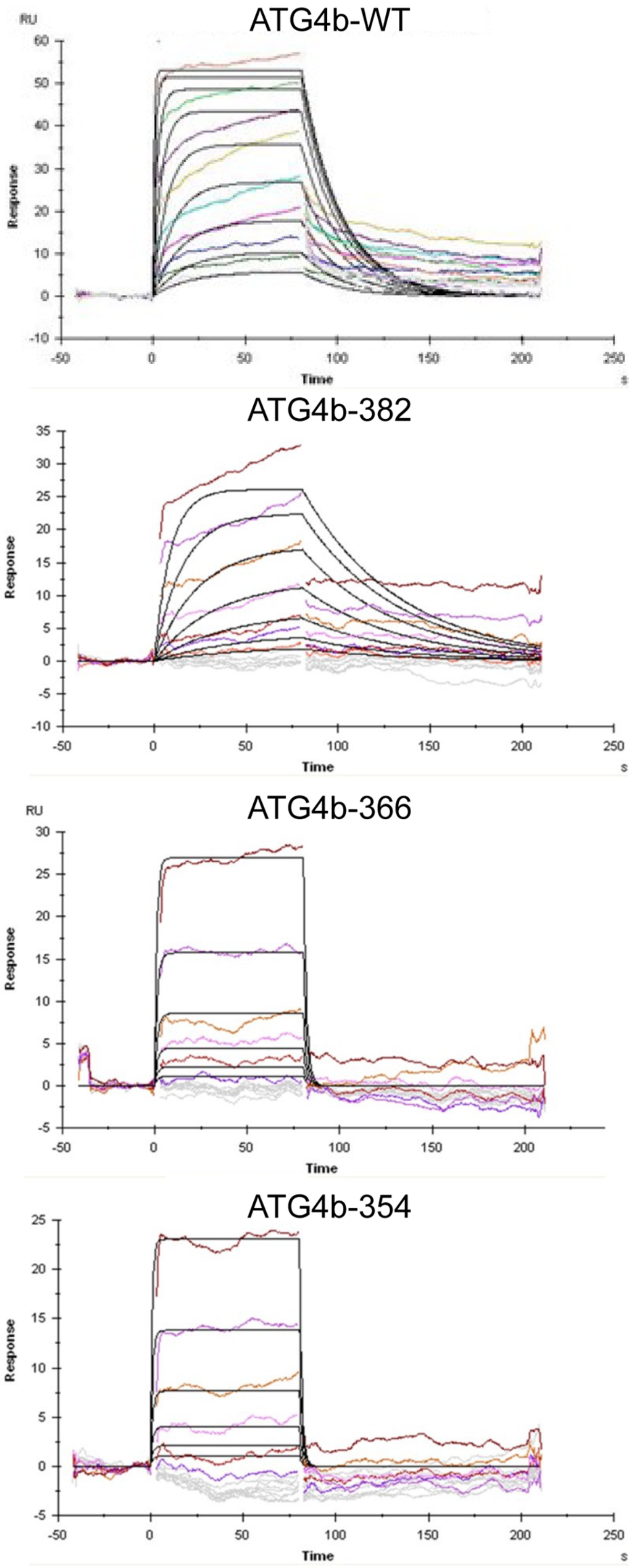

Figure S6. Representative SPR sensorgrams of ATG4b proteins binding to LC3B-I fit to a 1:1 kinetic binding model.

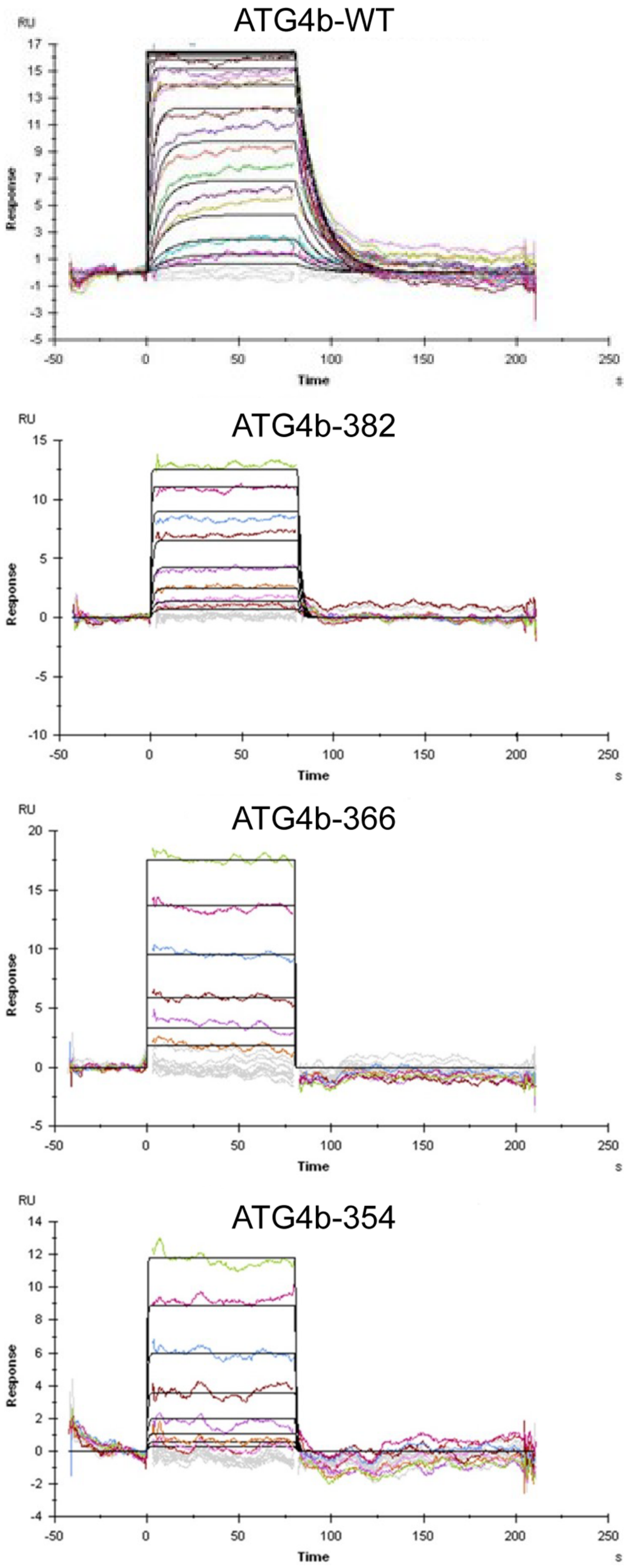

Figure S7. Representative SPR sensorgrams of ATG4b proteins binding to LC3B-115 fit to a 1:1 kinetic binding model.

Table S1. Expected masses and observed masses for LC3B constructs.

	Expected	Observed	*in vivo* biotinylation (%)
LC3B-115	18068.5	18068.5	91%
Biotinylated LC3B-115	18295.5	18294.5	
LC3B-116	18196.6	18196.5	75%
Biotinylated LC3B-116	18423.6	18423.0	
LC3B-118	18426.8	18426.5	50%
Biotinylated LC3B-118	18653.8	18625.5	
LC3B-I	18631.0	18631.0	27%
Biotinylated LC3B-I	18858.0	18858.0	
Pro-LC3B	19189.8	19189.5	64%
Biotinylated pro-LC3B	19416.0	19416.5	

Table S2. Kinetic rate constants for binding of LC3B-I and LC3B-115 to ATG4b truncations.

	**LC3B-I**			**LC3B-115**		
	k_{on} ($M^{-1}S^{-1}$)	k_{off} (S^{-1})	Kinetic K_D (μM)	k_{on} ($M^{-1}S^{-1}$)	k_{off} (S^{-1})	Kinetic K_D (μM)
**ATG4b-WT**	$8.6{\times}10^5 \pm 9.7{\times}10^4$	0.046 ± 0.02	0.084 ± 0.047	$5.5{\times}10^6 \pm 8.4{\times}10^5$	0.045 ± 0.007	0.0084 ± 0.0019
**ATG4b-382**	No fit	No fit	No fit	$1.9{\times}10^5 \pm 6.5{\times}10^4$	0.82 ± 0.28	4.4 ± 0.6
**ATG4b-366**	$1.7{\times}10^4 \pm 1.7{\times}10^4$	0.59 ± 0.22	87 ± 11	$1.4{\times}10^5 \pm 3.8{\times}10^4$	1.3 ± 0.5	9.5 ± 1.4
**ATG4b-354**	$3.6{\times}10^3 \pm 9.7{\times}10^4$	0.51 ± 0.27	140 ± 74	$7.5{\times}10^4 \pm 3.8{\times}10^4$	1.1 ± 0.4	15.7 ± 8.4

Table S3. Primers used to make ATG4b constructs.

Primer Name	Primer sequences (5'-3')
P1	GCTCGGCGCGCCTGCAGGTCGACATGGACGCAGCTACTCTGACCTAC
P2	GGTTTCTTTACCAGACTCGAGTCAAAGGGACAGGATTTCAAAGTCTTC
P3	GGTTTCTTTACCAGACTCGAGTCACTGCTCCACCAGCTCAAACATGGG
P4	GGTTTCTTTACCAGACTCGAGTCAGTCGAAGAATCTTTCCAGTCGCTC
P5	GGTTTCTTTACCAGACTCGAGTCAGTTCAGGACGTCGGGGCAGGCCAG

Table S4. Primer combinations for ATG4b pET-15b vector constructs.

No.	Primer combination	Constructs	Notes
1	P1 + P2	ATG4b-WT	Full length
2	P1 + P3	ATG4b-354	C-terminal truncation
3	P1 + P4	ATG4b-382	c-terminal truncation
4	P1 + P5	ATG4b-366	c-terminal truncation

Table S5. Primers used to make LC3B constructs.

Primer Name	Primer sequence (5'-3')
P6	ATGCCGTCGGAGAAGACCTTCAAG
P7	CACTGACAATTTCATCCCGAACGTC
P8	CCCGAACGTCTCCTGGGAGGCACAG
P9	CGTCTCCTGGGAGGCACAGACCATG
P10	GGAGGCACAGACCATGTACAGGAATC

Table S6. Primer combinations for LC3B N-His-Avi vector constructs.

No.	Primer combination	Constructs	Notes
1	P6 + P7	Pro-LC3B	Pro-LC3B full length
2	P6 + P8	LC3B-I	LC3B-I full length
3	P6 + P9	LC3B-118	Ubiquitin core + 3 residues
4	P6 + P10	LC3B-115	Ubiquitin core

2. Supplementary sequences

LC3B-115: HisTag—TEV cleavage sequence—AviTag—LC3B(1-115) (Vector backbone: pET-15b)

```
MGSSHHHHHHSSGENLYFQGHMSGLNDIFEAQKIEWHEGGGSMPSEKTFKQRRTFEQRVEDVRLIREQH
PTKIPVIIERYKGEKQLPVLDKTKFLVPDHVNMSELIKIIRRRLQLNANQAFFLLVNGHSMVSVSTPIS
EVYESEKDEDGFLYMVCAS*
```

LC3B-116: HisTag—TEV cleavage sequence—AviTag—LC3B(1-116) (Vector backbone: pET-15b)

```
MGSSHHHHHHSSGENLYFQGHMSGLNDIFEAQKIEWHEGGGSMPSEKTFKQRRTFEQRVEDVRLIREQH
PTKIPVIIERYKGEKQLPVLDKTKFLVPDHVNMSELIKIIRRRLQLNANQAFFLLVNGHSMVSVSTPIS
EVYESEKDEDGFLYMVCASQ*
```

LC3B-118: HisTag—TEV cleavage sequence—AviTag—LC3B(1-118) (Vector backbone: pET-15b)

```
MGSSHHHHHHSSGENLYFQGHMSGLNDIFEAQKIEWHEGGGSMPSEKTFKQRRTFEQRVEDVRLIREQH
PTKIPVIIERYKGEKQLPVLDKTKFLVPDHVNMSELIKIIRRRLQLNANQAFFLLVNGHSMVSVSTPIS
EVYESEKDEDGFLYMVCASQET*
```

LC3B-I: HisTag—TEV cleavage sequence—AviTag—LC3B(1-120) (Vector backbone: pET-15b)

```
MGSSHHHHHHSSGENLYFQGHMSGLNDIFEAQKIEWHEGGGSMPSEKTFKQRRTFEQRVEDVRLIREQH
PTKIPVIIERYKGEKQLPVLDKTKFLVPDHVNMSELIKIIRRRLQLNANQAFFLLVNGHSMVSVSTPIS
EVYESEKDEDGFLYMVCASQETFG*
```

Pro-LC3B: HisTag—TEV cleavage sequence—AviTag—LC3B(1-125) (Vector backbone: pET-15b)

```
MGSSHHHHHHSSGENLYFQGHMSGLNDIFEAQKIEWHEGGGSMPSEKTFKQRRTFEQRVEDVRLIREQH
PTKIPVIIERYKGEKQLPVLDKTKFLVPDHVNMSELIKIIRRRLQLNANQAFFLLVNGHSMVSVSTPIS
EVYESEKDEDGFLYMVCASQETFGMKLSV*
```

ATG4b-WT: HisTag—TEV cleavage sequence—AviTag—AT4b(1-393) (Vector backbone: pET-15b)

```
MGSSHHHHHHSSGENLYFQGHMSGLNDIFEAQKIEWHEGGGSMDAATLTYDTLRFAEFEDFPETSEPVW
ILGRKYSIFTEKDEILSDVASRLWFTYRKNFPAIGGTGPTSDTGWGCMLRCGQMIFAQALVCRHLGRDW
RWTQRKRQPDSYFSVLNAFIDRKDSYYSIHQIAQMGVGEGKSIGQWYGPNTVAQVLKKLAVFDTWSSLA
VHIAMDNTVVMEEIRRLCRTSVPCAGATAFPADSDRHCNGFPAGAEVTNRPSPWRPLVLLIPLRLGLTD
INEAYVETLKHCFMMPQSLGVIGGKPNSAHYFIGYVGEELIYLDPHTTQPAVEPTDGCFIPDESFHCQH
PPCRMSIAELDPSIAVGFFCKTEDDFNDWCQQVKKLSLLGGALPMFELVEQQPSHLACPDVLNLSLDSS
DVERLERFFDSEDEDFEILSL*
```

ATG4b-382: HisTag—TEV cleavage sequence—AviTag—AT4b(1-382) (Vector backbone: pET-15b)

MGSSHHHHHHSSGENLYFQGHMSGLNDIFEAQKIEWHEGGGSMDAATLTYDTLRFAEFEDFPETSEPVW
ILGRKYSIFTEKDEILSDVASRLWFTYRKNFPAIGGTGPTSDTGWGCMLRCGQMIFAQALVCRHLGRDW
RWTQRKRQPDSYFSVLNAFIDRKDSYYSIHQIAQMGVGEGKSIGQWYGPNTVAQVLKKLAVFDTWSSLA
VHIAMDNTVVMEEIRRLCRTSVPCAGATAFPADSDRHCNGFPAGAEVTNRPSPWRPLVLLIPLRLGLTD
INEAYVETLKHCFMMPQSLGVIGGKPNSAHYFIGYVGEELIYLDPHTTQPAVEPTDGCFIPDESFHCQH
PPCRMSIAELDPSIAVGFFCKTEDDFNDWCQQVKKLSLLGGALPMFELVEQQPSHLACPDVLNLSLDSS
DVERLERFFD*

ATG4b-366: HisTag—TEV cleavage sequence—AviTag—AT4b(1-366) (Vector backbone: pET-15b)

MGSSHHHHHHSSGENLYFQGHMSGLNDIFEAQKIEWHEGGGSMDAATLTYDTLRFAEFEDFPETSEPVW
ILGRKYSIFTEKDEILSDVASRLWFTYRKNFPAIGGTGPTSDTGWGCMLRCGQMIFAQALVCRHLGRDW
RWTQRKRQPDSYFSVLNAFIDRKDSYYSIHQIAQMGVGEGKSIGQWYGPNTVAQVLKKLAVFDTWSSLA
VHIAMDNTVVMEEIRRLCRTSVPCAGATAFPADSDRHCNGFPAGAEVTNRPSPWRPLVLLIPLRLGLTD
INEAYVETLKHCFMMPQSLGVIGGKPNSAHYFIGYVGEELIYLDPHTTQPAVEPTDGCFIPDESFHCQH
PPCRMSIAELDPSIAVGFFCKTEDDFNDWCQQVKKLSLLGGALPMFELVEQQPSHLACPDVLN*

ATG4b-354: HisTag—TEV cleavage sequence—AviTag—AT4b(1-354) (Vector backbone: pET-15b)

MGSSHHHHHHSSGENLYFQGHMSGLNDIFEAQKIEWHEGGGSMDAATLTYDTLRFAEFEDFPETSEPVW
ILGRKYSIFTEKDEILSDVASRLWFTYRKNFPAIGGTGPTSDTGWGCMLRCGQMIFAQALVCRHLGRDW
RWTQRKRQPDSYFSVLNAFIDRKDSYYSIHQIAQMGVGEGKSIGQWYGPNTVAQVLKKLAVFDTWSSLA
VHIAMDNTVVMEEIRRLCRTSVPCAGATAFPADSDRHCNGFPAGAEVTNRPSPWRPLVLLIPLRLGLTD
INEAYVETLKHCFMMPQSLGVIGGKPNSAHYFIGYVGEELIYLDPHTTQPAVEPTDGCFIPDESFHCQH
PPCRMSIAELDPSIAVGFFCKTEDDFNDWCQQVKKLSLLGGALPMFELVEQ*

CFP-LC3B-YFP: HisTag—CFP—LC3B—YFP

MHHHHHHMVSKGEELFTGVVPILVELDGDVNGHKFSVSGEGEGDATYGKLTLKFICTTGKLPVPWPTLV
TTLTWGVQCFSRYPDHMKQHDFFKSAMPEGYVQERTIFFKDDGNYKTRAEVKFEGDTLVNRIELKGIDF
KEDGNILGHKLEYNYISHNVYITADKQKNGIKANFKIRHNIEDGSVQLADHYQQNTPIGDGPVLLPDNH
YLSTQSALSKDPNEKRDHMVLLEFVTAAGITLGMDELYKGSMPSEKTFKQRRTFEQRVEDVRLIREQHP
TKIPVIIERYKGEKQLPVLDKTKFLVPDHVNMSELIKIIRRRLQLNANQAFFLLVNGHSMVSVSTPISE
VYESEKDEDGFLYMVYASQETFGMKLSVKLMVSKGEELFTGVVPILVELDGDVNGHKFSVSGEGEGDAT
YGKLTLKFICTTGKLPVPWPTLVTTFGYGLQCFARYPDHMKQHDFFKSAMPEGYVQERTIFFKDDGNYK
TRAEVKFEGDTLVNRIELKGIDFKEDGNILGHKLEYNYNSHNVYIMADKQKNGIKVNFKIRHNIEDGSV
QLADHYQQNTPIGDGPVLLPDNHYLSYQSALSKDPNEKRDHMVLLEFVTAAGITLGMDELYK*
